# Dynamics of human take and animal predation on sea turtle nests in Northwest Costa Rica

**DOI:** 10.7717/peerj.12925

**Published:** 2022-04-26

**Authors:** Janie L. Reavis, Daniela Rojas-Cañizales, Carmen Mejías-Balsalobre, Isabel Naranjo, Randall Arauz, Jesse F. Senko

**Affiliations:** 1 School of Life Sciences, Arizona State University, Tempe, AZ, United States of America; 2 Rescue Center for Endangered Marine Species (CREMA), San Jose, Costa Rica; 3 Grupo de Trabajo en Tortugas Marinas del Golfo de Venezuela (GTTM-GV), Maracaibo, Venezuela; 4 Red de Investigadores Actuando por el Medio Ambiente (RIAMA), Madrid, Spain; 5 Fins Attached Marine Research and Conservation, Colorado Springs, CO, United States of America; 6 School for the Future of Innovation in Society, Arizona State University, Tempe, AZ, United States of America

**Keywords:** Nesting beach, Sea turtle, Conservation, Human take, Wildlife extraction, Depredation, Olive ridley

## Abstract

Many conservation projects relocate sea turtle eggs to hatcheries to protect the sea turtle nests from the anthropogenic and natural threats they face in the early stages of development. The Rescue Center for Endangered Marine Species (CREMA) manages four sea turtle conservation projects on the nesting beaches of the Southern Nicoya Peninsula in Costa Rica, where the predominant nesting activity is from olive ridley turtles (*Lepidochelys olivacea*). Two of these nesting projects are based in Costa de Oro and San Miguel, which are adjacent beaches divided by an estuary. In this study, we compared the dynamics and rates of human and animal predation of nests prior to being relocated to the hatchery on both nesting beaches from 2012 to 2018. We hypothesized that human take and animal predation were compensatory threats, meaning that lower human take may result in higher animal predation, and vice versa, resulting in a similar number of nests lost to predation overall. We discuss the community-based conservation programs on both beaches, one of which has been monitored since 1998 (San Miguel) and the other of which has been monitored since 2012 (Costa de Oro). We found that Costa de Oro exhibited high rates of human take with up to 51% of nests being extracted per season, which has decreased since the conservation project was established. Human take was significantly higher than animal predation on both beaches and human take was significantly higher in Costa de Oro. While San Miguel exhibited higher animal predation, the difference was not statistically significant. Higher depredation by animals corresponded to higher overall nest abundance on both beaches. We were unable to find evidence that human take or animal predation increased in the absence of the other threat, suggesting a lack of compensatory effects of predation. Our findings support further analysis of animal predation and a continuation of patrol-based conservation efforts as well as community outreach to attempt to merge cultural values with sea turtle conservation.

## Introduction

Anthropogenic threats impact marine wildlife globally and include climate change, fisheries bycatch, direct take, habitat degradation, pollution and pathogens, and utilization of wildlife products (Wallace et al., 2011; Gelcich et al., 2014; Kovacs et al., 2012). Sea turtles have been depredated for centuries due to their reliance on nearshore and terrestrial habitats for reproduction (Frazier, 2003; Dawson et al., 2017). Therefore, nesting beaches are considered areas of vulnerability for sea turtles because adult turtles and the eggs they deposit can face either direct take by humans or depredation by natural predators (Heithaus et al., 2008).

In Costa Rica, sea turtles have been an important food resource for coastal communities for millennia (Frazier, 2005). Today, although the extraction of eggs is illegal in Costa Rica with the exception of the Ostional Wildlife Refuge in accordance with Wildlife Conservation Law 6919 (Campbell, Haalboom & Trow, 2007; Valverde et al., 2012), it still occurs on many beaches throughout the country (Hart, Gray & Stead, 2013; Santidrián Tomillo et al., 2008; Pheasey et al., 2020). On the other hand, nest predation by animals has also been studied in Costa Rica in the past (Fowler, 1979). Today, hatching success on some nesting beaches in the country is more threatened by animal predation than by human take (Barquero-Edge, 2013).

To mitigate anthropogenic and natural impacts that sea turtle nests face in early stages and increase overall hatching rates, many sea turtle conservation programs employ hatcheries as a last resort to relocate eggs from beaches to protected areas where they can be incubated until the hatchlings are subsequently released (Mejías-Balsalobre & Bride, 2016; Naro-Maciel, Mrosovsky & Marcovaldi, 1999). However, hatcheries are resource-intensive and often unable to relocate all nests for protection. Other nesting programs on beaches that incur more predation by non-human predators than take by humans have employed wire netting over nests *in situ* to prevent exhumation by predators or a natural beach hatchery, which are both less resource-intensive than an artificial hatchery (Barquero-Edge, 2013; Rees, Tzovani & Margaritoulis, 2002). Non-human predators can include reptiles, birds, crustaceans, insects, and mammals, so one solution will likely not deter all possible predators (Phillott, 2020). Therefore, context of the primary threats on each nesting beach is imperative to choose the most effective conservation strategy.

The Rescue Center for Endangered Marine Species (CREMA by its Spanish acronym) is an NGO that has maintained long-term nesting beach conservation projects in Costa Rica since 1998 (Rojas-Cañizales et al., 2022). On the nesting beaches monitored by CREMA on the Nicoya Peninsula, the main threat to sea turtle nests is generally human take of sea turtle eggs (Pheasey et al., 2020; Viejobueno Muñoz & Arauz, 2015; Viejobueno, Adams & Arauz, 2011). Therefore, eggs are relocated to hatcheries within the first 12 h after oviposition. This has resulted in the release a total of over 170,000 sea turtle hatchlings while supporting local communities and promoting public awareness for marine conservation *via* community involvement and educational outreach (CREMA). The majority of nesting turtles are olive ridleys (*Lepidochelys olivacea*), but leatherback (*Dermochelys coriacea*), green (*Chelonia mydas*), and hawksbill turtles (*Eretmochelys imbricata*) occasionally nest on these beaches (Viejobueno, Adams & Arauz, 2011). Since 1998 when the first project began, CREMA has sought to protect nesting sea turtles as well as eggs and hatchlings while also spreading awareness among the community about the value of sea turtles, both for the environment and socioeconomic benefits through ecotourism. While human take is the primary threat on CREMA’s beaches, sea turtle nests approximately 150 miles south on the Osa Peninsula are more threatened by non-human depredation, likely due to less accessible infrastructure (Barquero-Edge, 2013; Drake, 1996). It is therefore important to understand the dynamics of human take and animal depredation of sea turtle nests in Costa Rica and elsewhere. For example, in the presence of high take by humans, does less animal predation occur? Likewise, in the absence of human take, does animal predation increase? The extent to which these potential compensatory effects may influence hatchling output is important, but many conservation programs only closely monitor the nests relocated to *ex situ* hatcheries as opposed to *in situ* nests (Chacón-Chaverri & Eckert, 2007).

In this study we aimed to understand the relationship between human take and animal predation of sea turtle nests managed by conservation programs on two nesting beaches in Northwest Costa Rica. We hypothesized that the effects of human take and animal predation will be compensatory, meaning that total nests predated on both beaches will be similar despite high variation between human take and animal predation. It is possible that the absence of one threat leaves more nests vulnerable to the other or that non-human predators, especially mammals, may be deterred by human presence near sea turtle nests. Specifically, we compared the combined impact of animal depredation and human take on both beaches to understand potential compensatory effects of predation dynamics and assess the most common predators of sea turtle nests.

## Methods

### Study sites

**Figure 1:**
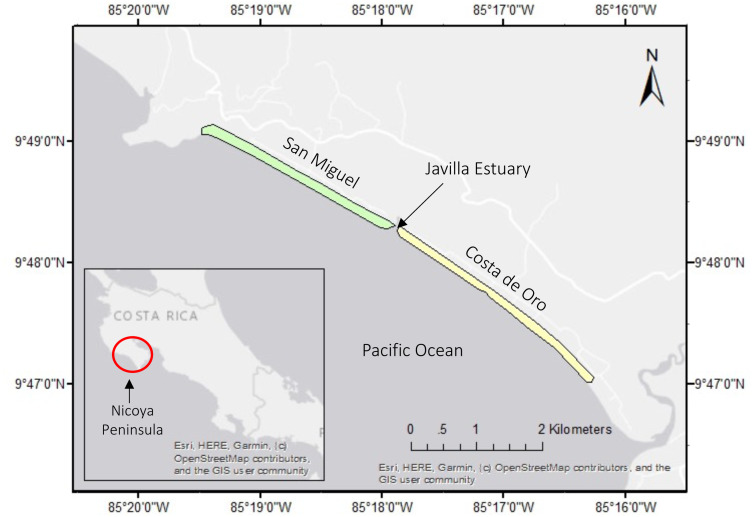
San Miguel and Costa de Oro nesting beaches on the northwest coast of Costa Rica. Inset map indicates the Nicoya Peninsula, where these beaches are located.

Our study includes two adjacent nesting beaches : San Miguel (9°48′40.83″N, 85°18′36.49″W) and Costa de Oro (9°47′58.04″N, 85°17′24.28″W) located on the Southern Nicoya Peninsula in Costa Rica and currently monitored by CREMA (Fig. 1). The nesting beaches are divided by the Javilla Estuary. Costa de Oro is the southernmost beach spanning 4.6 km, while San Miguel is in the northwest and is 2.5 km long (Rojas-Cañizales et al., 2020). The beaches are located on the outskirts of the corresponding towns, Costa de Oro and San Miguel, each of which has a year-round occupancy of about 50 residents (anonymous, pers. comm., 2017). The nesting activity for olive ridley sea turtles on this Pacific coast occurs from July to November (Dornfeld et al., 2015).

### Data collection

We compiled all nesting events recorded in Costa de Oro and San Miguel from June 2012 to December 2018 by CREMA. The conservation project in San Miguel began in 1998, while the project in Costa de Oro began in 2012, but we only evaluated data beginning in 2012 for consistency in data recording. Data were collected during nightly patrols, which lasted six to eight hours, and morning censuses on each beach during the seven nesting seasons. The monitoring was conducted at different hours to maximize the encounters with sea turtles that typically vary with the tides (Frazer, 1983). We recorded the date, location, species of turtle, successful nesting event categories (nest, predated or poached) and unsuccessful nesting event categories (aborted nest and false crawl), and type of predation (*i.e.,* animal or human). Predated nests were determined by the presence of animal footprints and broken eggshells around the nest (Leighton, Horrocks & Kramer, 2011), and nests were determined to have been depredated by humans when they exhibited at least two of the following signs: human footprints, holes made with a stick (presumably to locate the nest), or absence of eggs in a fully formed nest chamber (Troëng, Chacón & Dick, 2004). Nests partially depredated were documented as such and left *in situ* and checked during the morning census when late nesting events were also recorded. Complete nests were relocated to a hatchery within 12 h of oviposition. Additionally, in the 2018 nesting season, we recorded the predator species for depredated nests, when known. All research was approved by Ministerio de Ambiente y Energía permits and protocols under the following permit numbers: 2012- No ACT-OR-DR-148, 2013- No ACT-OR-DR-104-13, 2014- No ACT-OR-DR-105-16, 2015- No ACT-OR-DR-145-17, 2016- No ACT-OR-DR-112-16, 2017- No ACT-OR-DR-109-17, and 2018- No ACT-OR-DR-006-19.

### Data analysis

Statistical analyses were performed on nesting data recorded from July 2012 to December 2018. The analyses were performed using proportions, which were calculated by dividing the number of nests recorded as predated by animals or taken by humans each month by the total number of nests per month. We used a Shapiro–Wilks test to test normality of data and a Levene’s test to test equal variance, and subsequent tests were chosen based on these results. A two proportions *z*-test was then used to test the null hypothesis that the proportion of depredated nests (defined as proportion of animal depredation plus proportion of nests taken by humans) was not significantly different between San Miguel and Costa de Oro. Combined effects of beach and threat (human take or animal depredation) on the proportion of total nests depredated were analyzed using a Kruskal-Wallis rank sum test followed by a Wilcoxon rank sum test. We used a simple linear regression to test the relationship between number of nests taken by humans and number of nests depredated by animals. We also used a simple linear regression to test the relationship between depredation by animals and overall nest abundance on both beaches. All statistical tests were performed in R version 3.6.1, and hypotheses were tested with a significance level of 0.05. Averages are reported with standard deviation.

## Results

### Predation dynamics

**Figure 2:**
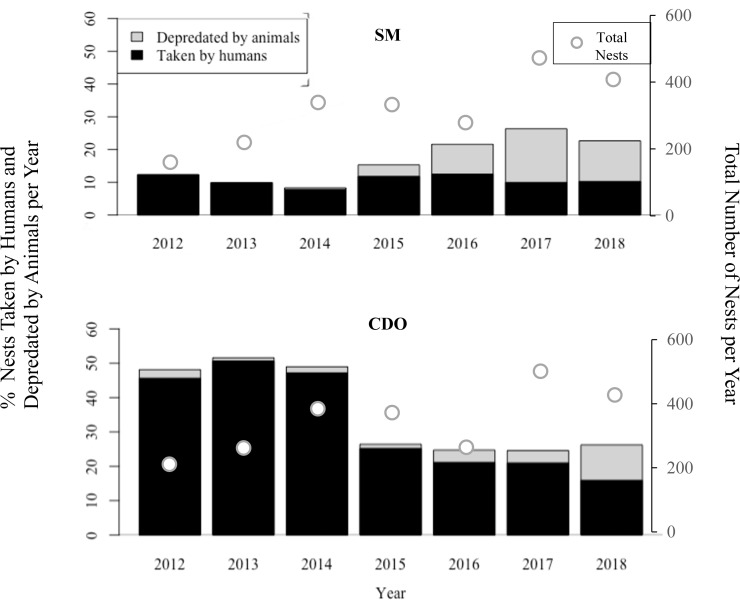
Percent of olive ridley turtle (*L. olivacea*) nests depredated by animals and humans seasonally, along with total nests, from 2012 to 2018, in San Miguel (SM) and Costa de Oro (CDO).

Predation of olive ridley nests by combined anthropogenic and natural threats were higher in Costa de Oro, with the exception of the 2017 nesting season when both Playa San Miguel and Playa Costa de Oro had 25% (*n* = 132 and *n* = 116, respectively) of nests depredated by a combination of animal predation and human take (Fig. 2). Pressure from human take was consistently higher in Costa de Oro (exceeding that of San Miguel by as little as 6% in 2018 (*n* = 21) and as much as 41% (*n* = 85) in 2014 (Wilcoxon rank sum: *p* < 0.001). Human take in San Miguel varied less year-to-year, ranging from approximately 8–13%, than human take in Costa de Oro (16–51%). Meanwhile, the proportion of nests depredated by animals in San Miguel exceeded depredation from 2015 to 2018 in Costa de Oro (Fig. 2).

**Figure 3:**
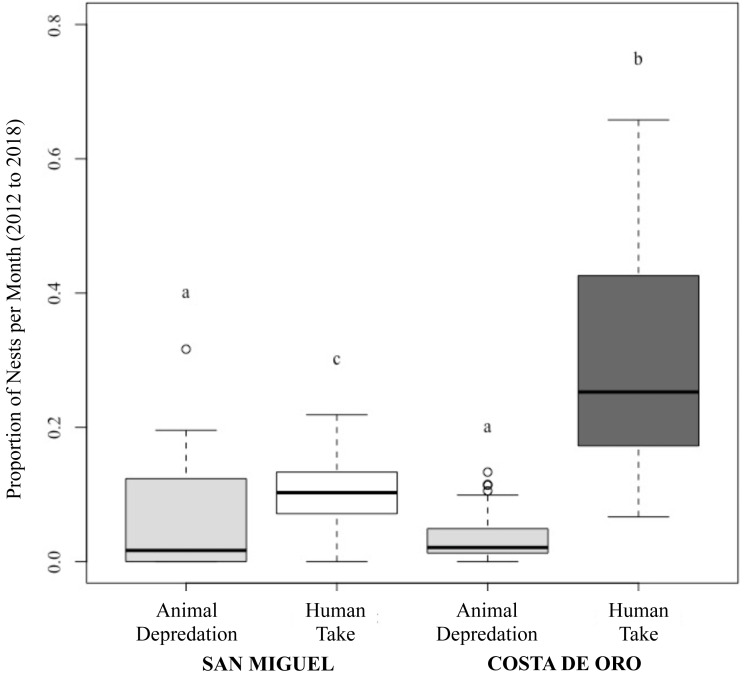
Proportion of olive ridley turtle (*L. olivacea*) nests depredated by animals and humans per month in San Miguel and Costa de Oro between 2012 and 2018. Significant differences shown by letters (a, b, c).

While the number of total nesting events in San Miguel and Costa de Oro were relatively similar year to year, the proportion of nests depredated by animals and humans were significantly different (Two proportions *z*-test: *z* = 22.76, *df* = 57, *p* < 0.001; Fig. 3). Although the proportion of human take was significantly different between the beaches (Wilcoxon rank sum: *p* < 0.001; Fig. 3), we were unable to reject the null hypothesis that proportion of animal depredation does not differ between locations (Wilcoxon rank sum: *p* = 0.857; Fig. 3). The total nests depredated, both by humans and animals, was also greater in Costa de Oro than in San Miguel (*t*-test: *t* = 2.772, *df* = 6, *p* = 0.032). A simple linear regression showed no significant relationship between number of nests taken by humans and number of nests depredated by animals (simple linear regression: *t* = −0.92, *df* = 56, *R*^2^ = 0.01, *p* = 0.36). Another simple linear regression indicated that higher depredation by animals corresponded to higher overall nest abundance on both beaches (simple linear regression: *t* = 2.58, *df* = 12, *R*^2^ = 0.36, *p* = 0.02).

### Variation over time

There was no clear decline in nest predation *via* combined anthropogenic and natural threats in San Miguel (Fig. 2). The proportion of nests threatened by human take decreased every year since 2013 in Costa de Oro and every year since 2016 in San Miguel (Fig. 2). In 2012 and 2013, 46% (*n* = 73) and 50% (*n* = 111) of sea turtle nests were taken by humans in Costa de Oro, respectively, while in 2017 and 2018, take decreased to 21% (*n* = 99) and 15% (*n* = 65) during the nesting season. Total nesting event numbers varied little over time from 2012 to 2016, the average number of nesting events for Playa San Miguel and Playa Costa de Oro was 369 ± 80 and 306 ± 80, respectively. Meanwhile, San Miguel had 538 nests recorded in 2017 and 530 in 2018, while Costa de Oro had 502 and 429 nesting events in 2017 and 2018, respectively (Fig. 2).

### Animal predator species

**Figure 4:**
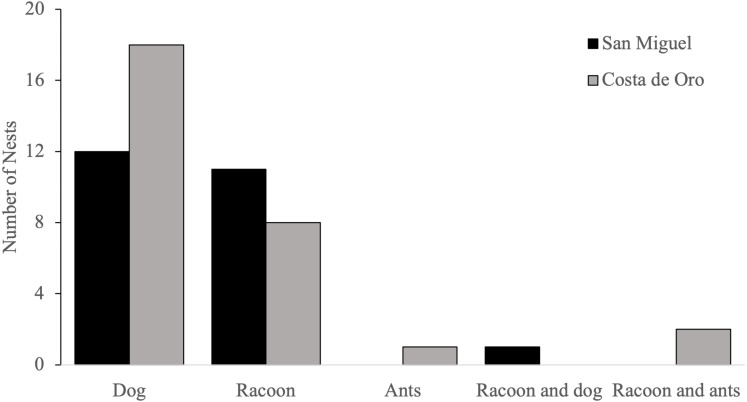
Number of nests predated by each animal in San Miguel and Costa de Oro during the 2018 nesting season.

Domestic dogs (*Canis familiaris*) were the most frequently encountered predator, accounting for 58% (*n* = 31) of olive ridleys nests predated by animals during the 2018 nesting season, followed by raccoons (*Procyon lotor*), which accounted for 42% (*n* = 22) of depredated nests (Fig. 4). Both species depredated the same nest once, while ants (Formicidae) 2% (*n* = 1) were the main predator of one nest and were present in two nests that had been previously depredated by raccoons.

## Discussion

Despite the prevalence of one threat over another on these adjacent beaches, the difference in animal predation between beaches was not as large as the difference in human take; thus, Costa de Oro has more total nests lost due to the combined threats (Fig. 2). Additionally, there was no significant negative relationship between nests taken by humans and nests depredated by animals. Therefore, we did not find any data to support the hypothesis of compensatory effects of predation, although predation dynamics during the last two years may indicate the commencement of a compensatory trend. The hypothesis that these threats, animal depredation and human take, are inversely related is currently not supported by our data. It is important to note that this study was focused on animal depredation and human take within 12 h of a nest being laid, as the majority of nests were relocated to the hatchery on the same night they were laid, which protected them from depredation, human take, and other threats such as nest inundation after the first night (Sari & Kaska, 2017).

Although sea turtle eggs have been utilized historically for food by inhabitants of coastal regions worldwide (Frazier, 2003), the proportion of eggs taken by humans varies from one community to another, relating to differing cultural values and economic conditions (Mejías-Balsalobre et al., 2021). We found that human take was higher in Costa de Oro than in San Miguel. The time difference between the conservation projects could be the reason for the decrease in egg extraction over the time, as San Miguel opened 14 years before Costa de Oro. We did not report data from the San Miguel project’s inception in 1998 due to changes in data recording, but human take has decreased from 1998 to 2010 and currently is drastically decreased since then (Viejobueno, Adams & Arauz, 2011). Instead, we compared the consistently low level of human take in San Miguel in recent years to the change in human take in Costa de Oro with the implementation of a nesting beach conservation program.

Human take in Costa de Oro has decreased consistently from 2014 to 2018 with a 2-year delay after the conservation program’s inception in 2012. This indicates that the project in Costa de Oro is still developing and has already made progress in decreasing human take. Similar trends are evident as a result of nesting beach patrols and hatcheries on other beaches of the Costa Rican Pacific coast, where human take decreased from 85% of nests to 10% (James & Melero, 2015). James & Melero (2015) demonstrated a drastic decrease of human take from 2005, the beginning of the hatchery program, to 2006, with the decreased take persisting each year thereafter. Despite the hatchery opening in 2012 in Costa de Oro, human take did not clearly decrease until 2015. This may be due to a lack of saturation of community conservation efforts (Theodossopoulos, 1997). An important difference between the two beaches is that the conservation efforts in San Miguel were started by the community prior to CREMA’s involvement, whereas the Costa de Oro program was not. Another reason for the delay in effectiveness could be that people who take sea turtle eggs in Costa de Oro tend to come from other communities on motor bikes (anonymous, pers. comm., 2017), meaning community efforts in Costa de Oro may be limited if the threat is external. It is also possible that human take decreasing on one beach results in a redistribution of human take on other beaches, resulting in a subsequent increase of take somewhere else. Unfortunately, we do not have data to confirm either of these theories. The take and consumption of olive ridley eggs has been a custom in Central American coastal communities for generations (Hart, Gray & Stead, 2013; Hope, 2002). The motivation for egg consumption varies from economic benefits to nutritional purposes, however, many communities are more likely to support conservation efforts involving the residents and bringing economic revenue (Senko et al., 2011; Mejías-Balsalobre et al., 2021). Realistically, with such varied causes, one conservation strategy may not be a blanket solution for all (Hampshire et al., 2004).

Depredation of sea turtle nests is a natural occurrence on nesting beaches (Fowler, 1979). A study by Leighton and colleagues showed that human presence on nesting beaches deterred mongooses, a common diurnal predator of hawksbill sea turtles, from the nesting habitat (Leighton, Horrocks & Kramer, 2010). Similar reasoning led us to suspect that increased human presence on Costa de Oro and San Miguel, whether it be for conservation purposes or human take, might deter non-human predators. However, in 2018 in Costa de Oro and San Miguel the primary non-human predators of sea turtle nests on both beaches were human-introduced mammals, which are a common predator to coastal wildlife on beaches worldwide (Fontaine, Gimenez & Bried, 2011; Aguirre-Muoz et al., 2008;  Ruiz-Izaguirre et al., 2015). Depredation on both Costa de Oro and San Miguel has increased since 2015 alongside human population growth and subsequent domestic dog population growth (CREMA, pers. comm., 2018). Human presence has likely not affected domestic dog activity on these nesting beaches because the dogs in this area are familiar with humans. Many dogs in the community are free to roam at all hours, including nighttime when sea turtles most often nest (Fritsches & Warrant, 2013). Other studies have recommended restricting the movement of domestic canines from 8 pm to dawn or identifying problematic canines *via* radio collars and focusing restriction on those that commonly predate nests (Ruiz-Izaguirre et al. 2015; Sepúlveda et al., 2015). However, the potential success of such a strategy would depend on the willingness of community members and cultural factors (Ruiz-Izaguirre & Eilers, 2012).

Raccoons were the second most common predator of olive ridley eggs on both beaches, while ants were only a contributing predator in three nests, making them the least common predator. Raccoons have been reported as predators on sea turtle nesting beaches in North and Central America, even warranting predator removal programs in extreme cases (Engeman et al., 2010). Although the Nicoya Peninsula is within the natural home range of raccoons (Helgen & Wilson, 2005), they are known to aggregate near human populations (Prange, Gehrt & Wiggers, 2004). Consequently, predation by raccoons and potentially other mesocarnivores may also be increased by human presence.

Depredation was also impacted by total nest numbers during a season. Our data indicates that higher abundance of nests may attract more predators, especially when the increased scent of nests would likely be more detectable (Kajobe & Roubik, 2006). A higher occurrence of nesting would also enhance predators’ ability to develop a search image (Ruzicka & Conover, 2012), which might explain why the two years of greatest depredation in San Miguel were also the years with the highest number of nests overall.

Considering there was more depredation in recent years despite more established conservation efforts, we observed no clear effect of conservation efforts on depredation by animals thus far. Before the implementation of nesting beach conservation programs, human take of sea turtles and, to a lesser extent their eggs, has been shown to cause declines in some sea turtle populations (García, Ceballos & Adaya, 2003). Many conservation programs have decreased the threat of humans to nesting sea turtles, leading to speculation about other threats such as non-human predation and beach erosion (Santidrián Tomillo et al., 2007; Tripathy & Rajasekhar, 2009). In fact, other beaches in Costa Rica are threatened more by non-human predation while being less affected by human take (Barquero-Edge, 2013). Other wildlife that faces the threat of human take also face other threats that may not be directly caused by humans, such as non-human predation, food depletion, or changing landscape composition (Chapron et al., 2008). This can cause a dilemma in prioritizing the allocation of conservation action and funds. For example, black rhino populations were heavily exploited in the 1970’s by human take, and now that protection against humans has been increased, researchers have discovered possible mortality from predation by spotted hyenas (Sillero-Zubiri & Gottelli, 1991). Similarly, giraffe populations in Tanzania that previously suffered from human predation may now suffer from a depletion of palatable plants in the region (Strauss et al., 2015). Like our study, researchers were concerned that with decreased human predation, other threats might now increase. However, these studies as well as others show non-human threats having additive effects rather than compensatory effects, which is also clear in San Miguel where the variation in human take is minimal, but the addition of non-human predation causes overall nest loss to double in some years (Fig. 2; Ramesh et al., 2017).

## Conclusion

The San Miguel conservation program has resulted in a successful reduction of human take, whereas Costa de Oro still has consistently higher numbers for human take of sea turtle nests. We attribute this largely to the longevity of the San Miguel project compared to the Costa de Oro project; these projects have been run by CREMA and the local community since 1998 and 2012, respectively. The community in San Miguel is accustomed to and supportive of the nesting beach protection and associated ecotourism, but it is possible that Costa de Oro is lacking community saturation in terms of outreach, education, and economic benefits. Therefore, an evaluation of the two beaches after the Costa de Oro program had been established for 10 years or more, with consistent data collection and recording protocol, would be more representative of the predation dynamics on the two beaches with well-established conservation programs.

We suggest the continuation of hatchery and patrol-based conservation efforts along with community-based programs informing residents of the importance of reducing illegal take of sea turtle eggs in both San Miguel and Costa de Oro as well as implementation of nest relocation on other nesting beaches facing threats such as animal depredation, human take, beach erosion, and nest inundation. Hatchery programs also protect eggs from animal predation following relocation. Long-term analysis of the nesting population on both beaches would be beneficial in the future to elucidate the effects of both threats and conservation efforts (Strauss et al., 2015). Additionally, we recommend measures to reduce depredation by domesticated dogs, which may include neutering of pets in the community along with maintaining the nesting beach protocol requiring researchers to record predator species in the case of depredated nests, thus allowing long-term monitoring of depredation.

## Supplemental Information

10.7717/peerj.12925/supp-1 Supplemental Information 1 Raw nesting beach data for San Miguel and Costa de Oro from 2012 to 2018 Each row represents one nesting event

## Additional Information and Declarations

### Data Deposition

The following information was supplied regarding data availability: The raw nesting beach data including every nesting event that was recorded during our study period (June 2021 to December 2018) is available in the Supplemental File.
